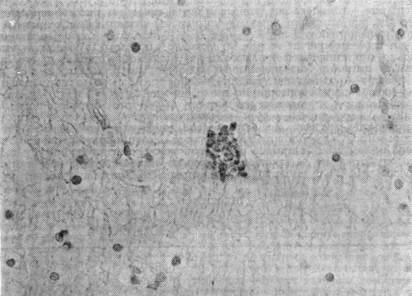# ERRATUM

**DOI:** 10.1590/S0004-282X1993000100002err

**Published:** 2021-06-24

In the manuscript “Histopathological and immunohistochemical study of the brain and heart in the chronic cardiac form of Chagas' disease”, DOI: 10.1590/S0004-282X1993000100002, published in the Arq Neuropsiquiatr 1993;51(1):8-15, on page 4.

**Where it reads:**

**Figure 2:**
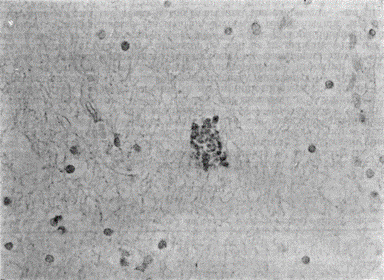

**Figure 3:**
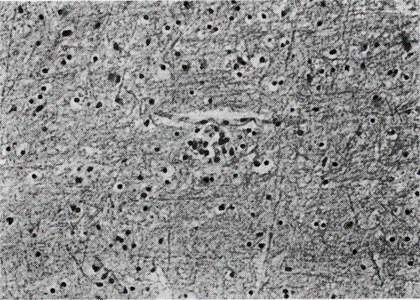

**It should read:**

**Figure 2:**
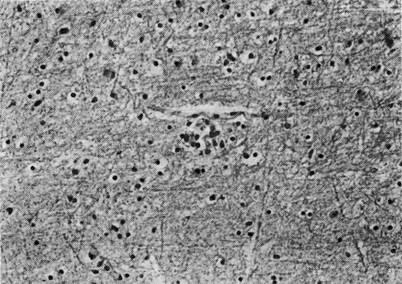

**Figure 3:**